# Bioinformatic analysis reveals an evolutional selection for DNA:RNA hybrid G-quadruplex structures as putative transcription regulatory elements in warm-blooded animals

**DOI:** 10.1093/nar/gkt781

**Published:** 2013-09-02

**Authors:** Shan Xiao, Jia-yu Zhang, Ke-wei Zheng, Yu-hua Hao, Zheng Tan

**Affiliations:** State Key Laboratory of Biomembrane and Membrane Biotechnology, Institute of Zoology, Chinese Academy of Sciences, Beijing 100101, P. R. China

## Abstract

Recently, we reported the co-transcriptional formation of DNA:RNA hybrid G-quadruplex (HQ) structure by the non-template DNA strand and nascent RNA transcript, which in turn modulates transcription under both *in vitro* and *in vivo* conditions. Here we present bioinformatic analysis on putative HQ-forming sequences (PHQS) in the genomes of eukaryotic organisms. Starting from amphibian, PHQS motifs are concentrated in the immediate 1000-nt region downstream of transcription start sites, implying their potential role in transcription regulation. Moreover, their occurrence shows a strong bias toward the non-template versus the template strand. PHQS has become constitutional in genes in warm-blooded animals, and the magnitude of the strand bias correlates with the ability of PHQS to form HQ, suggesting a selection based on HQ formation. This strand bias is reversed in lower species, implying that the selection of PHQS/HQ depended on the living temperature of the organisms. In comparison with the putative intramolecular G-quadruplex-forming sequences (PQS), PHQS motifs are far more prevalent and abundant in the transcribed regions, making them the dominant candidates in the formation of G-quadruplexes in transcription. Collectively, these results suggest that the HQ structures are evolutionally selected to function in transcription and other transcription-mediated processes that involve guanine-rich non-template strand.

## INTRODUCTION

G-quadruplex, a four-stranded secondary structure formed by guanine-rich (G-rich) nucleic acids, is gaining increasing attention owing to its potential role in physiological and pathological processes (1–4). DNA G-quadruplexes have recently been shown to exist in the genome of living mammalian cells (5). Putative G-quadruplex sequences (PQS) are prevalent in the human genome, which count to ∼37 000 copies in known genes (6,7). Formation of G-quadruplex in DNA affects a number of physiological processes associated with DNA, to mention a few examples, telomere extension (8,9), DNA tracking (10), methylation (11) and genome instability (12). Because of its abundance in promoter regions (13), a more general function of G-quadruplex in a genome is believed to play a role in transcription regulation. This functionality is first demonstrated for the intramolecular G-quadruplex structure upstream of the P1 promoter of C-MYC that controls the transcriptional activation of the gene (14) and later for the G-quadruplex structures in many other genes (15–21). Bioinformatic searches of genomic DNA revealed that PQS are enriched around transcription start sites (TSS) in a variety of organisms, providing a strong support to a general role of G-quadruplex structures in transcription (6,7,22–31).

**Figure 1.**
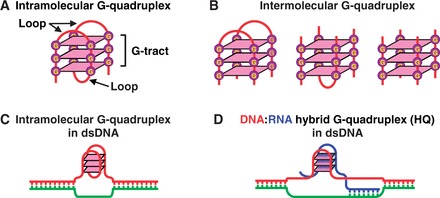
Examples of G-quadruplexes. (**A**) An intramolecular G-quadruplex of three G-quartet layers. (**B**) Intermolecular G-quadruplexes composed of two, three and four nucleic acid strands, respectively. (**C**) An intramolecular G-quadruplex in dsDNA. (**D**) An DNA:RNA hybrid quadruplex (HQ) in dsDNA.

G-quadruplexes can be grouped into two simple categories, i.e. intramolecular and intermolecular structures, according to the number of nucleic acid strands involved in the assembly of the structures. A single nucleic acid strand bearing four G-tracts can fold into an intramolecular G-quadruplex containing a stack of guanine quartets (G-quartet) linked by three loops (Figure 1A). On the other hand, intermolecular G-quadruplex can form by acquiring four G-tracts from multiple nucleic acid strands (Figure 1B). To date, investigation on G-quadruplexes of genomic sources has been focused on intramolecular G-quadruplexes (Figure 1C). While the presence of G-quadruplex structures in living cells has recently been detected (5), the biogenesis of G-quadruplexes in cells remains largely unclear. Recently, we reported that transcription of double-stranded DNA (dsDNA) readily produces DNA:RNA hybrid G-quadruplexes (HQ) by G-tracts from both the non-template DNA strand and the nascent RNA transcript (Figure 1D). In addition, we found that such HQ formation in turn modulates transcription under both *in vitro* and *in vivo* conditions. We further showed that putative HQ-forming sequences (PHQS) are present in >97% of human genes and their number correlate with the transcriptomal profiles in human tissues (32). These results suggest that HQ structures have a fundamental role and could be a more prevalent form of G-quadruplexes in genome.

To further explore the physiological implication and characterize the occurrence of PHQS motifs in genomes, we carried out genome-wide analysis to organisms whose genomic data are currently available in the Ensembl genes database. Here we show that PHQS is present in much greater prevalence and abundance than the PQS. Like the PQS, PHQS motifs are also concentrated near TSS. HQ formation requires G-tracts from the non-template strand. In accordance with this, PHQS motifs exhibited preferential enrichment on the non-template strand. Our data suggest that this strand bias might be selected by a mechanism based on the capability of PHQS to form HQ. Analysis across different organisms illustrates that a negative selection of PHQS occurred in the genomes of metazoa and pisces. In contrast, a positive selection began to merge in amphibians and PHQS became constitutional in genes in warm-blooded animals. Collectively, these results suggest that HQ structures are evolutionally selected to function in transcription regulation and other transcription-mediated processes that involve the transcription of DNA with guanine-rich non-template strand, such as immunoglobulin class switching, recombination, genomic instability and replication initiation.

## MATERIALS AND METHODS

### Gene sequences

Sequences of protein-coding genes and their upstream flanking region were downloaded in fasta format, respectively, along with their IDs from the Ensembl genes database (release 68, except *Mustela putorius furo*, which was from release 69) via the BioMart (version 0.7) interface (http://www.ensembl.org/) by selecting protein-coding in the Gene type filter and Unspliced (Gene) in the Attribute/Sequences panel. Only unique results were downloaded.

### Sequence analysis

**Figure 2.**
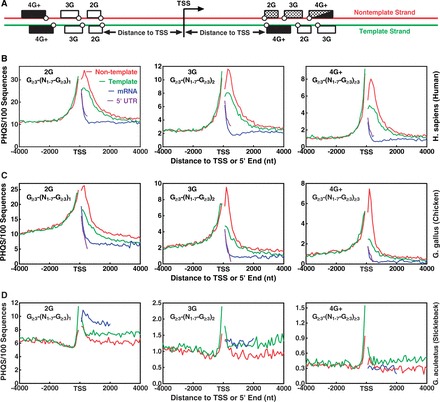
Distribution of putative DNA:RNA HQ-forming sequences (PHQS) with different numbers of G-tracts in the ±4 kb region of TSS. (**A**) Scheme of PHQS distribution. Dotted block: motifs capable of forming HQ (the 4G+ may also form non-hybrid intramolecular G-quadruplexes); filled block: capable of forming intramolecular G-quadruplex; open block: unable to form either HQ or intramolecular G-quadruplex. Open circle indicates the coordinate of G-tract (the first guanine). (**B**) Human, 22 058 genes, (**C**) Chicken, 16 736 genes, (**D**) Stickleback, 20 787genes. Matching pattern is indicated in each panel. Color designation for the curves in the first panel of human also applies to all the other panels (same in the whole paper). Frequency was normalized to the number of sequences and expressed as the number of occurrences in 100 sequences within a 100-nt window.

PHQS was identified with a home-made Perl program (Supplementary Figure S1, original transcript and a standalone executable file are provided) developed using the Active Perl 5.14.2 (downloaded from www.activestate.com/activeperl) under the Windows OS. The program used a pattern-matching code G{3,}(.{1,7}?G{3,}){1,} to detect the sequences G_≥3_-(N_1__–7_-G_≥3_)_≥1_, where G denoted guanine and N denoted any nucleotide, including G. The use of non-greedy quantifiers for loops while the rest operators were greedy by default ensured that G-tracts would not be ignored or treated as loop. Putative G-quadruplex sequences (PQS) were identified in the same way, but using the pattern-matching code G{3,}(.{1,7}?G{3,}){3,} that detects sequences G_≥3_-(N_1__–7_-G_≥3_)_≥3_. Each match returned the matched sequence, its coordinate (the position of the first guanine relative to TSS, Figure 2A) and gene ID. The motifs found were grouped into four categories designated 1G, 2G, 3G and 4G+ in which they contained 1, 2, 3 and ≥4 G-tracts, respectively. They were then sorted into 100 nt bins based on their coordinates to obtain their frequency distribution. Because the pattern matching was within the whole sequence rather in windows of defined size, motifs of more than four G-tracts are identified as single hits and no overlaps would occur. The search of each sequence file generates two tab delimited plain text files containing information on each found PHQS, their occurrence distribution and statistical summary of the PHQS motifs on both the non-template and template strands, respectively, within a designated searching range (Supplementary Figure S1 and Supplementary Table S1). The files can be opened in Excel or similar software for viewing and further processing. Isolated G_3_ tracts were analyzed similarly using the pattern-matching code G{2,}(.{1,7}?G{2,}){0,} that identifies all motifs with one or more G-tracts of two or more consecutive guanines, connected by loops of 1–7 nucleotides. Any found motifs that had more than one G-tract, or G-tract with size ≠ 3 were discarded. The remaining motifs are single G_3_ tracts isolated from other G-tracts by more than seven nucleotides. They were then processed in the same way as the PHQS.

### Masking of regulatory motifs

Motif masking for CpG islands and G-rich transcription factor binding sites (TFBSs) was conducted using the software MotifLab version 1.07 (33). A list of IDs for the protein-coding genes in human (GRCh37.p11) was obtained from the Ensembl genes database (release 72). A CpG island BED file for human was downloaded using the Table Browser of the University of California Santa Cruz (UCSC) Genome Browser (http://genome.ucsc.edu/) from the assembly hg19, track CpG islands, in BED format. Three BED files for the G-rich TFBS, EGR1, MAZ and SP1, was downloaded using the UCSC Table Browser from the assembly hg19, track Uniform TFBS, respectively. The gene list was imported into the MotifLab to download the correspondent DNA sequences and the BED file for the motifs was imported to mask the sequences. Masked sequences were then analyzed by the homemade Perl script (Supplementary Figure S1). Masking of heterogeneous nuclear ribonucleoproteins (hnRNP) A and H motifs was conducted using a home-made Perl script that searched for the motifs in the DNA sequences and converted them to N's as described (25).

### HQ formation in *in vitro* transcription

dsDNA carrying a T7 promoter and an indicated downstream G-core was prepared by overlap extension polymerase chain reaction. Transcription was carried out essentially as previously described (32) at 37°C for 1 h using 50 nM dsDNA in a 10 µl volume of 40 mM Tris–HCl (pH 7.9) buffer containing 2 U/µl T7 polymerase (Fermentas, USA), 50 mM KCl, 40% (w/v) polyethylene glycol (PEG) 200, 8 mM MgCl_2_, 10 mM dithiothreitol (DTT), 2 mM spermidine, 2 mM nucleoside triphosphate (NTP) and 0.5 U/µl pyrophosphatase, inorganic (Fermentas, USA). After transcription, the sample was diluted with equal volume of stop solution containing 40% (w/v) PEG 200, 50 mM KCl, 1 µM competitive DNA (5′-GAAATTAATACGACTCACTATA-3′, double-stranded), 0.8 µg/µl RNase A and 0.4 U/µl RNase H, followed by a incubation at 37°C for 1 h. The reaction was terminated by addition of 1/25 vol of 0.5 M EDTA and 1/20 vol of 2% sodium dodecyl sulfate. The DNA was then resolved on a 10% polyacrylamide gel containing 75 mM KCl and 40% (w/v) PEG 200, at 4°C, 8 V/cm, in 1× tris-borate-EDTA (TBE) buffer containing 75 mM KCl. Resolved DNA was detected by the fluorescence of carboxyfluorescein (FAM) dye labeled at the 5′ end of the non-template strand using a Typhoon 9400 phosphor imager (GE Healthcare, USA).

## RESULTS

### Strand-biased enrichment of PHQS in TSS-flanking region

To survey the occurrence of PHQS in genomes, we carried out computational searches in the protein-coding genes in species in the Ensembl genes database. The search algorithm found all motifs that match the sequence pattern G_≥3_-(N_1__–7_-G_≥3_)_≥1_; that is, two or more G-tracts of three or more consecutive guanines, connected by loops of 1–7 nucleotides. Because the formation of HQ in transcription requires a minimum of two G-tracts from the DNA strand, our searching pattern was adopted from the one G_≥3 _-(N_1-7_-G_≥3_)_≥3_ that has been used in searches for PQS in genomes in the original (6,7) and many later works (13,22–31,34–37) by simply reducing the minimal number of G-tracts from four to two. The PHQS motifs were then grouped into four categories designated 1G, 2G, 3G and 4G+, which contain 1, 2, 3 and ≥4 G-tracts, respectively. It should be noted that the 4G+ motifs are also capable of forming intramolecular DNA G-quadruplex. The 1G group contained long G-tracts that satisfy the pattern G_≥3 _-(N_1-7_-G_≥3_)_≥1_ and can be clarified into one or more of the other three categories. For example, a G_15_ can be regarded either as a 2G sequence of G_7_-L_1_-G_7_ or as a 4G sequence of G_3_-L_1_-G_3_-L_1_-G_3_-L_1_-G_3_ or others, where L_1_ designates a 1-nt (G) loop. Because of their small amount (<1% of PHQS in human genes) and multiple clarifications, they were not used for further frequency analysis. Figure 2B–D present the results obtained from human, chicken and stickleback, which gives the occurrence frequency of PHQS in the ±4 kb region centered at TSS on both the non-template and template strands.

Similar to the PQS motifs (13,23,26,38), the PHQS motifs were also enriched in the region adjacent to TSS in human and chicken, mostly within the immediate 1 kb region (Figure 2B and C). The enrichment is present on both sides of TSS and on both the template and non-template DNA strands. Because the PHQS motifs in the region upstream of TSS and in the template strand downstream of TSS are in principle unable to form HQ, they must be selected by mechanisms that are irrelevant to HQ. However, the distribution of PHQS showed a greater occurrence in the non-template strands than in the template strands downstream of TSS, which is also similar to that of the PQS motifs (13,23,26,38). In addition, this strand bias is not present in the region upstream of TSS that is not transcribed. These two facts suggest that the strand bias toward PHQS motifs on the non-template strand is specifically associated with transcription and selected by an additional mechanism(s). Comparison with mRNA showed that the PHQS motifs near the TSS were largely removed after splicing (Figure 2B and C). Therefore, most of the PHQS motifs are intended to function in transcription and/or pre-mRNA.

### PHQS strand bias is positively selected in warm-blooded animals

**Figure 3.**
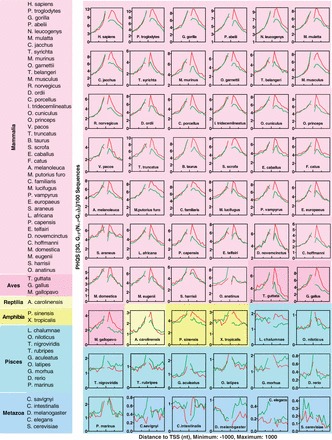
Biased selection of PHQS in 60 species in the Ensembl database. Each panel shows the occurrence of PHQS with three G-tracts (3G) in the non-template (red curve) and template (green curve) strands within the ±1-kb region centered at TSS. Similar distribution pattern and strand bias were also present for PHQS with two and four G-tracts (2G and 4G+) as in Figure 2. The species list is ordered according to the species tree provided on the Ensembl Web site to reflect the order of evolution.

**Figure 4.**
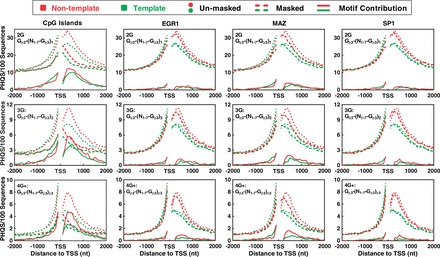
Contribution of CpG islands and G-rich TFBSs (EGR1, MAZ and SP1) to the enrichment and strand bias of PHQS near TSS. DNA sequences were searched for PHQS motifs before (dotted curves) and after (dashed curves) masking the correspondent motif. The difference between the two searches gave the contribution of the motif. Results were processed and expressed as in Figure 2. Red and green curves indicate non-template and template DNA strand, respectively.

If the strand bias for PHQS is selected for a biological function, it should be conserved across related species. To trace its evolutional selection, we searched for PHQS in the genomes of all the species currently available in the Ensembl database. It can be seen that the species in the mammalian, avian, reptilian and amphibian categories showed biased positive selection for PHQS on the non-template strand, downstream of TSS for the 2G, 3G (Figure 3) and 4G+ motifs (Supplementary Figure S2). However, such a strand bias was not obvious for the species in the pisces and metazoan categories. More precisely, a reversed negative selection could be noticed in most of these species in which the occurrence of PHQS was higher in the template than in the non-template strand. One exception was the *Latimeria chalumnae* in the pisces, which also displayed a higher occurrence in the non-template than in the template strand, like the two of the amphibian species. The selection in those three species was disturbed by random noise in the background. This might reflect an evolutional transition from lower to higher organisms in the selection for PHQS.

Overall, an enrichment of PHQS near TSS and strand bias toward PHQS on the non-template strand began to show up in amphibians, and they became a general feature in the warm-blooded animals (aves and mammalia). In contrast, lower organisms showed much lower occurrences of PHQS than higher organisms. The reservation of a strand-biased enrichment of PHQS across the warm-blooded species argues that PHQS motifs are evolutionally selected.

### An independent mechanism for the selection of PHQS strand bias

Promoters overlap TSS (39) and often harbor G-rich regulatory elements, such as CpG islands (40), TFBSs (24,25) and recognition sites for posttranscription (41,42) and translation (43) regulation. There are two possibilities that may account for the strand bias of PHQS. It could be selected either to produce G-rich RNA to form HQ or serve as recognizing elements in pre-mRNA/mRNA (41,42). The small number of PHQS motifs that remained at the 5′ end of spliced mRNA may represent those in the 5′ UTR region, based on their distribution overlap (Figure 2B and C, Blue and pink curves), which may play other functions in translation (43). The G-rich elements that function specifically in pre-mRNA/mRNA are expected to contribute to the strand bias. At least, those recognized by the hnRNP are specific to the non-template strand (41,42), thus should contribute to the PHQS strand bias. To evaluate the contribution of G-rich regulatory elements, we determined the occurrence of PHQS in human with several G-rich elements masked. They include CpG island, EGR1, MAZ, SP1 and hnRNP binding sequences (hnRNP).

Figure 4 shows the results obtained for the CpG island and three G-rich TFBSs: EGR1, MAZ and SP1 motifs, respectively. The masking of the CpG islands significantly reduced the occurrence of PHQS on both the non-template and template strand (dashed versus dotted curve), but the strand bias downstream of TSS remained across all the three categories of PHQS motifs after the masking (red versus green dashed curves). The CpG islands obviously contributed to the strand bias of PHQS as indicated by a higher selection for them on the non-template than on the template strand (red versus green solid curves). Similar results were also obtained for the EGR1, MAZ and SP1 motifs with respect to their influence on the occurrence of PHQS and contribution to the strand bias, although in a reduced magnitude. These results indicated that CpG islands, TFBS and similar G-rich regulatory motifs provide a source for the enrichment of PHQS and they were differentially selected on the two DNA strands to promote HQ formation.

**Figure 5.**
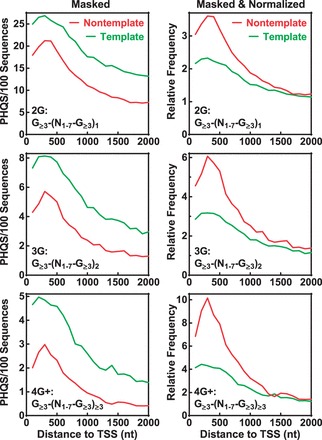
Contribution of hnRNP A and H to the enrichment and strand bias of PHQS downstream of TSS. Four G-rich motifs were masked in the following order: TAGGGT/A, GGGA, only on the non-template strand. Frequency is expressed as in Figure 2. Left panels: original data obtained after masking. Right panels: data obtained by normalizing the original data to the background (mean of the data points in the 3000–4000 nt region) of each curve. Red and green curves indicate non-template and template DNA strand, respectively.

Unlike the above elements, the G-rich motifs in RNA transcripts recognized by hnRNP A and hnRNP H is non-template specific. Therefore, the masking in this case was conducted only for the G-rich elements. This manipulation reversed the strand bias in the entire region for human (Figure 5, left panels). When compared with the template strand, however, it can be noticed that the occurrence frequency near TSS on the non-template strand is still much higher relative to the background level. To make a better comparison, we normalized the occurrence of PHQS to the background and this restored the stand bias (Figure 5, right panels). This result clearly shows that the enrichment of PHQS was preferentially promoted on the non-template than on the template strand near the TSS.

### PHQS strand bias correlates with the efficiency of PHQS to form HQ

**Figure 6.**
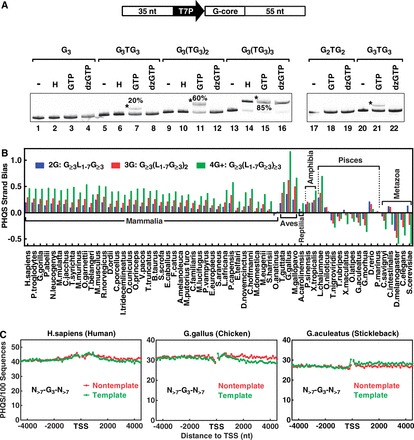
PHQS strand bias correlates with the potential of PHQS to form HQ. (**A**) Formation of stable HQ requires a minimum of two G_3_ tracts. DNA bearing the indicated G-core downstream of a T7 promoter (T7P) was subjected to no treatment (−), or a heat denaturation/renaturation (H), or transcription with T7 polymerase and GTP or dzGTP. HQ formation was detected by native gel electrophoresis. Asterisk indicates the band of DNA containing HQ and its amount as percent of the total DNA in the lane. (**B**) PHQS Strand Bias in 61 species in the Ensembl database. The species is ordered according to the species tree provided on the Ensembl Web site. (**C**) Occurrence of isolated G_3_ tracts in the ±4-kb region around TSS in human, chicken and stickleback. Red and green curves indicate non-template and template DNA strand, respectively.

In seeking a cause that leads to PHQS strand bias, it was noticed that the magnitude of the strand bias in human and chicken increases with the number of G-tracts (Figure 2B and C). Our previous experiments showed that HQ formation also increased with an increase in the number of G-tracts (32) (Figures 2C and 3B therein). This suggests that the strand bias might be selected by the capability of PHQS to form HQ. Because the sequences derived from the NRAS gene in our previous work varied in the size of both the loops and G-tracts, and loop composition, we determined more stringently the dependence of HQ formation on the number of G-tracts by experiments. Co-transcriptional formation of HQ was analyzed in dsDNAs (Figure 6A, scheme) bearing a G-core of different G-tracts with single-T loops. HQ formation in these DNAs was detected by native gel electrophoresis after a posttranscription digestion with RNase A and H to remove all the RNAs except those in HQ. DNA carrying a G-quadruplex migrates at slower rate than the same DNA containing no G-quadruplex (32,44). As is shown in Figure 6A, no HQ was detected in the DNA containing only one G_3_ under any condition (lanes 1–4). For the DNAs containing two or three G_3_, however, HQ was observed when the transcription was carried out with normal GTP (lanes 7, 11). In contrast, no HQ was seen when the DNA was subjected to a heat denaturation/renaturation (lanes 6, 10) or transcribed with the GTP being substituted by 7-deaza-GTP (dzGTP) (lanes 8, 12), a GTP analog used to prevent RNA from participating in G-quadruplex formation (45). This fact indicates that these two DNAs were unable to form G-quadruplex by themselves, but needed the RNA to participate. It can be noted that the DNA with three G_3_ tracts formed more HQ (60%) than the DNA with two G_3_ tracts (20%). When the number of G_3_ tracts increased to four, more G-quadruplex formed (lane 15, 85%). Because this DNA alone was also able to form intramolecular G-quadruplex (lanes 14, 16), it is not known how much HQ formed in this DNA. The G-quadruplex structures detected were those that remained after the posttranscription processing and might not exactly reflect their amount formed during transcription. However, the higher HQ amount detected in the DNA with three G_3_ tracts than in that with two G_3_ tracts implies that more G-tracts led to more chance of HQ formation, which provides an intuitive explanation to a greater PHQS strand bias for the 3G than for the 2G motifs (Figure 2B and C).

To see if a higher strand bias is correlated with more G-tracts in the other warm-blooded species as in the human and chicken, we quantitated PHQS strand bias in all the species available in the Ensembl database using the following equation:

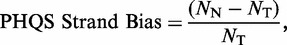

where *N*_N_ and *N*_T_ are the number of PHQS motifs in the non-template and template strand, respectively, within the 1 kb region downstream of TSS. This definition gives the relative excess of PHQS motifs in the non-template comparing with the template strand. In Figure 6B, it can be seen that all the warm-blooded animals, except the *Ornithorhynchus anatinus*, showed positive strand bias, implying a preferential selection for PHQS in the non-template strand in these species. More importantly, the strand bias all increases with an increase in the number of G-tracts. Even though the 4G+ motifs are able to form intramolecular G-quadruplex besides HQ, it showed the highest strand bias. This may indicate a competition of HQ formation against the intramolecular structure in these motifs. On the other hand, a negative selection seems to be present in the metazoa and pisces where PHQS occurrence in the majority of the organisms was suppressed in the non-template relative to the template strand as indicated by their negative strand bias values. In this case, a same dependence on the number of G-tracts, but in a reversed order, is also seen, i.e. motifs of more G-tracts correlate with stronger suppression or negative selection.

Our experimental results in Figure 6A (left panel) show that the formation of a HQ requires at least two tandem G-tracts in the non-template strand. A single G-tract, like the GGGA recognized by all the hnRNP H family proteins (41), is not able to form HQ in transcription, and masking the GGGA did not remove the strand bias of PHQS (Figure 5). We thought it might be of interest to see if a strand bias would also occur with such motifs that are unable to form HQ. We analyzed those G_3_ tracts that are isolated from other G-tracts in the non-template strand. Figure 6C gives the distributions of such orphan G_3_ motifs that are separated from any G_≥2_ by more than seven nucleotides. In accordance with their inability to form HQ, their distributions showed little strand bias as well as enrichment near TSS. Collectively, the results in Figures 2 and 6 suggest that the strand bias of PHQS has been developed based on the ability of PHQS to form HQ.

### PHQS is the dominant candidate for G-quadruplex formation in transcription

**Figure 7.**
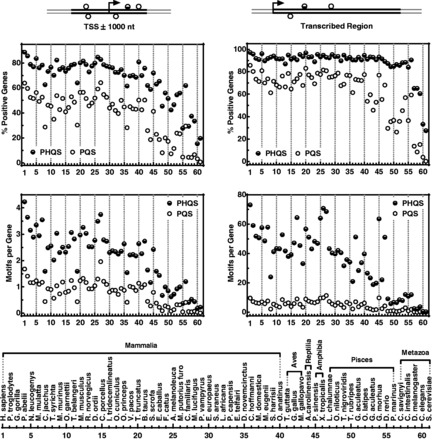
Occurrence of PHQS and PQS motifs in 61 species in the Ensembl database in the ±1-kb region around TSS (left panels) and transcribed region of genes (right panels). The species list is ordered according to the species tree provided on the Ensembl Web site.

Our previous work shows that HQ formation is a general feature associated with transcription of DNA bearing multiple G-tracts in the non-template strand. To survey the presence of PHQS in different genomes, we searched for PHQS in all the species in the Ensembl database and compared it with that of the PQS. Because the PHQS motifs are concentrated near the TSS and those near TSS are most relevant to transcription, we calculated their numbers within the ±1 kb region of TSS (Figure 7, left panels). We found that the PHQS is the dominant form of G-quadruplex-forming motifs in all the species. In mammalians, ∼80% of the genes carry PHQS, while ∼50% of the genes bear PQS. In lower species, the percentage of PHQS positive genes can be several times higher than that of the PQS. The PHQS showed a similar dominance when the average number of motifs per gene was calculated. In all the species, this value for the PHQS is averagely more than twice of that for the PQS. The abundance of both PHQS and PQS in lower species is dramatically lower than in higher species, but PHQS always maintained its dominance over PQS.

We also calculated the occurrence of PHQS and PQS within the transcribed region of the genes (Figure 7, right panels). In human, PHQS is present in >97% and PQS in >85% of the genes. This means nearly all genes in human are PHQS positive. For all the vertebrates, PHQS-positive genes are mostly between 90 and 100%. The average number of PHQS per human gene is >73 per gene, much greater than that of PQS, which is ∼10. All the other species have a lower PHQS load than human. The PQS load is mostly <10 per gene. The above statistics demonstrate that the putative HQ-forming sites were far more prevalent and abundant than the non-hybrid intramolecular G-quadruplex-forming sites in the eukaryotic organisms.

## DISCUSSION

As an extension of our previous work in which the co-transcriptional formation of HQ structures was revealed as a general phenomenon and characterized in details with experimental approaches (32), our present work presents a genome-wide analysis on the occurrence of PHQS motifs for the vertebrates and other eukaryotic species in the Ensembl database. Our analysis revealed the prevalence and abundance of PHQS in these species and pointed to an evolutional selection for PHQS. Although the analyses on the metazoa and pisces were with a limited number of data sets, our results suggested that the occurrence of PHQS motifs or, in other words, the formation of HQ is suppressed in these species as indicated by their negative PHQS strand bias. Starting from amphibians, the selection becomes positive as reflected by the positive PHQS strand bias and is reserved throughout the warm-blooded animals (Figure 6B). PHQS motifs have become constitutional in the genes of warm-blooded species (Figure 7). Interestingly, the *L.chalumnae*, which is thought to be an ancestor of amphibian, also shows significant positive PHQS strand bias as the amphibians.

Several lines of evidences (Figures 2 and 6) imply a connection of PHQS strand bias to the potential of the PHQS motifs to form HQ in transcription. The evolutional order of the strand bias (Figure 6B) seems to suggest that the selection is dependent of the living temperature of the species. The systematic selection of PHQS in mammalians and aves is associated with the ability of the organisms to maintain a constant body temperature. For the metazoa and pisces, the negative strand bias suggests that the HQ is physiologically deleterious; therefore, the occurrence of PHQS is suppressed (Figures 2, 3 and 7; Supplementary Figure S2), resulting in negative strand biases (Figure 6). The concentration of G-richness near the TSS as regulatory elements creates chances for HQ formation in transcription. The positive strand bias of PHQS in the warm-blooded animals implies that HQ structures selected are beneficial in these species with a stable body temperature.

HQ structures may function in two aspects. In general, lower organisms have fewer PHQS motifs per gene than higher organisms. In *Caenorhabditis elegans*, PHQS is only present in 33% of the genes, with an average of 0.6 PHQS per gene, in sharp contrast to the human genome. In *Saccharomyces cerevisiae*, PHQS is only found in 27.4% of the genes, with an average of 0.36 PHQS per gene. The large difference between the lower and higher organisms in the number of PHQS motifs per gene implied that the HQ might modulate transcription through different mechanisms. In the lower organisms, HQ may serve as recognition element, as intramolecular G-quadruplex does, that functions through binding with regulatory proteins (46). This functionality should also be present in higher organisms, but the universal presence and the large number of PHQS in warm-blooded species strongly suggested that the HQ structure has an additional, and perhaps more general, function independent of specific pathways. It is unlikely that a single human gene would use 73 HQs as recognition elements. Our previous work has shown that HQ modulates transcription under both *in vitro* and *in vivo* conditions, and the occurrence frequency of PHQS motifs in genes correlates with the transcriptional profiles in human tissues. As assumed in our previous work, HQ may regulate transcription in an intrinsic, direct and cost-effective way. We hypothesized that HQ structures may provide a general primary cis control at the root level of transcription to limit the expression potential of the host genes (32).

We expect that HQ should also have functionality in other processes that involve transcription. Strand-biased enrichment of guanine residues is featured in many physiologically important genomic elements, including immunoglobulin class switching sequences (47), prokaryotic (48) and mitochondrial (49) replication origins, the MAZ transcription termination element (50,51) and other transcribed genes (51,52). Transcription of G-rich DNA is a well-recognized source of genome instability, and it is often associated with a bias toward G-richness on the non-template strand (53–57). In principle, HQ formation may participate in cellular events that involve transcription of DNA with multiple G-tracts on the non-template strand. For example, transcription by the T7 RNA polymerase and mammalian RNA polymerase II is blocked when G-rich sequences are in the non-template strand, but not when they are in the template DNA strand, even in the presence of four G-tracts (58,59). Apparently, the formation of the HQ provides a reasonable explanation for the strand discrimination in those events because only the G-rich non-template can produce G-rich RNA transcripts, a prerequisite for HQ formation (Figure 1D).

The requirement of a minimum of two G-tracts instead of four allows PHQS motifs to occur at a much higher frequency than the PQS; thus, they are the dominant candidates for G-quadruplex formation in transcription in cells (Figure 7). The prevalence of PHQS motifs in genes and HQ formation associated with transcription potentially offers opportunity for manipulating the expression of nearly all genes by targeting HQ structures. On the other hand, this also brings an extreme challenge to the selectivity of G-quadruplex-interacting drugs. Indeed, it has been reported that administration of G-quadruplex ligands significantly affected the expression of a wide range of genes in human cells, in correlation with the presence of the predicted G-quadruplex sequences (60,61). Telomeric DNA tends to form intramolecular G-quadruplexes at the 3′ end of the DNA strand (62), and this inhibits its extension by both telomerase and the alternative lengthening of telomere (ALT) mechanism (9). Thus, stabilization of telomeric G-quadruplexes has long been pursued as an anticancer strategy (63). Previous investigations reported that G-quadruplex ligands induced senescence and telomere shortening in cancer cells (64–66). Given the prevalence of PHQS and PQS, this might suggest a combined effect of the drugs on telomeres and other G-quadruplex-bearing genes.

## SUPPLEMENTARY DATA

Supplementary Data are available at NAR Online.

## FUNDING

Ministry of Science and Technology of China [2013CB530802, 2012CB720601 and 2010CB945300]; National Science Foundation of China [30970617 and 21072189]. Funding for open access charge: Ministry of Science and Technology of China [2012CB720601].

*Conflict of interest statement*. None declared.

## Supplementary Material

Supplementary Data
