# Supplementary material for: Bioinformatic analysis reveals an evolutional selection for DNA:RNA hybrid G-quadruplex structures as putative transcription regulatory elements in warm-blooded animals
Source: Nucleic Acids Res. 2013 Sep 2;41(22):10379–90. doi: 10.1093/nar/gkt781 (PMC3905843; doi:10.1093/nar/gkt781)
Supplement: Supplementary Data [file supp_41_22_10379__index.html]

Bioinformatic analysis reveals an evolutional selection for DNA:RNA hybrid G-quadruplex structures as putative transcription regulatory elements in warm-blooded animals — Bioinformatic analysis reveals an evolutional selection for DNA:RNA hybrid G-quadruplex structures as putative transcription regulatory elements in warm-blooded animals — Supplementary Data 

# Bioinformatic analysis reveals an evolutional selection for DNA:RNA hybrid G-quadruplex structures as putative transcription regulatory elements in warm-blooded animals

## Supplementary Data

files

**Files in this Data Supplement:**

- Supplementary Data - pdf file
