# Supplementary material for: Bioinformatic analysis reveals an evolutional selection for DNA:RNA hybrid G-quadruplex structures as putative transcription regulatory elements in warm-blooded animals
Source: Nucleic Acids Res. 2013 Sep 2;41(22):10379–90. doi: 10.1093/nar/gkt781 (PMC3905843; doi:10.1093/nar/gkt781)
Supplement: Supplementary Data [file supp_gkt781_nar-00941-f-2013-File003.pdf]

Supplementary Data for

Bioinformatic analysis reveals an evolutional selection for DNA:RNA hybrid G-quadruplex structures as putative transcription regulatory elements in warm-blooded animals

Shan Xiao, Jia-yu Zhang, Ke-wei Zheng, Yu-hua Hao, Zheng Tan*

State Key Laboratory of Biomembrane and Membrane Biotechnology, Institute of Zoology, Chinese Academy of Sciences, Beijing 100101, P. R. China

*E-mail: z.tan@ioz.ac.cn; tanclswu@public.wh.hb.cn

1 supplementary table and 2 supplementary figures

Supplementary Table S1. Output report generated by the home-made Perl script of PHQS in genes in human chromosome Y in the 5100 nt range immediately downstream of TSS.

Human-Chr-Y.fa.G-rich.xls →

Figure S1

Perl package → (ZT-Find-PHQS-GUI.7z)

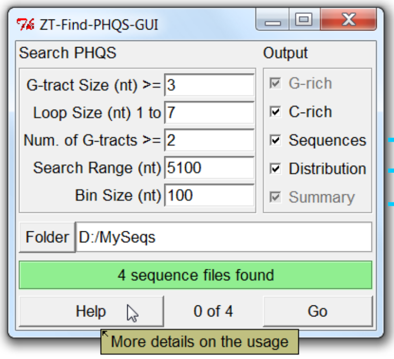

ID	Full Length	Match	Start	Length	Sequence	G-Tracts	Min Tract Size	Max Tract Size	Mini Loop Size	Max Loop Size
ENSG0000000457	41605	1	419	15	[GGGGGG]ccaaca[GGG]	2	3	6	6	6
ENSG0000000457	41605	2	531	21	[GGGGG]aa[GGGG]cc[GGG]c[G(	4	3	5	1	2
ENSG0000000457	41605	3	594	14	[GGGG]cccgcgc[GGG]	2	3	4	7	7
ENSG0000000457	41605	4	860	9	[GGG]cga[GGG]	2	3	3	3	3
ENSG0000000457	41605	5	878	7	[GGG]c[GGG]	2	3	3	1	1
ENSG0000000457	41605	6	916	16	[GGGGGG]atcct[GGGGG]	2	5	6	5	5
ENSG0000000457	41605	7	1885	16	[GGG]aggct[GGGG]a[GGG]	3	3	4	1	5
ENSG0000000457	41605	8	2880	30	[GGG]tgtcaat[GGG]atgtta[GGG]ta	4	3	3	5	7
ENSG0000000457	41605	9	5141	8	[GGGG]a[GGG]	2	3	4	1	1
ENSG0000000003	11322	1	90	16	[GGG]ct[GGG]ttaat[GGG]	3	3	3	2	5
ENSG0000000003	11322	2	226	19	[GGG]t[GGG]ca[GGG]acta[GGG]	4	3	3	1	4
ENSG0000000003	11322	3	343	29	[GGG]atga[GGGG]cttggta[GGG]a	4	3	4	4	7
ENSG0000000003	11322	4	497	11	[GGG]aa[GGGGGG]	2	3	6	2	2

Frequencies (Occurrence in 100 sequences within 100 nt window)

Bin	#ofSeqs	1G	2G	3G	4G+	ALL-G
100	22058	0.25388	30.1977	8.58645	5.49914	44.5371
200	22057	0.26296	31.5047	10.0422	7.39448	49.2043
300	22037	0.37664	34.3241	11.6259	8.03195	54.3586
400	22018	0.30884	31.8739	11.3498	7.58016	51.1127
500	21982	0.30479	30.5341	10.4449	7.04667	48.3305
600	21919	0.33304	27.6244	8.75952	5.65719	42.3742

Summary of searching (of 22058 sequences, with a mean length of 61696.72 nt)

G-tracts	1G	2G	3G	4G+	All-G
Positive Sequences	1784	21139	15967	11554	21311
% Positive Sequences	8.09	95.83	72.39	52.38	96.61
Total PHQS found	1978	173735	46336	25315	247364
Mean PHQS/Sequence	0.09	7.88	2.1	1.15	11.21
Mean PHQS/kb Sequence	0	0.13	0.03	0.02	0.18

Figure S1. User interface of the home-made Perl program and the output report it generated by searching for PHQS in human genes. The report consists of three sections (from top to bottom at right side) providing information on each PHQS, occurrence frequency, and statistical summary of the PHQS motifs, respectively. Search range 5100 bp downstream of TSS. To use the Perl package (Perl script, compiled stand-alone executable file and sample sequence files) attached, save the attachment, extract the files in the package into a folder using the 7-Zip free software (www.7-zip.org) and see instruction in the package. Windows OS users can use the program by double clicking on ZT-Find-PHQS-GUI.exe without installation of Perl. Installation of Perl is required to use the original Perl script ZT-Find-PHQS.pl.

Figure S2

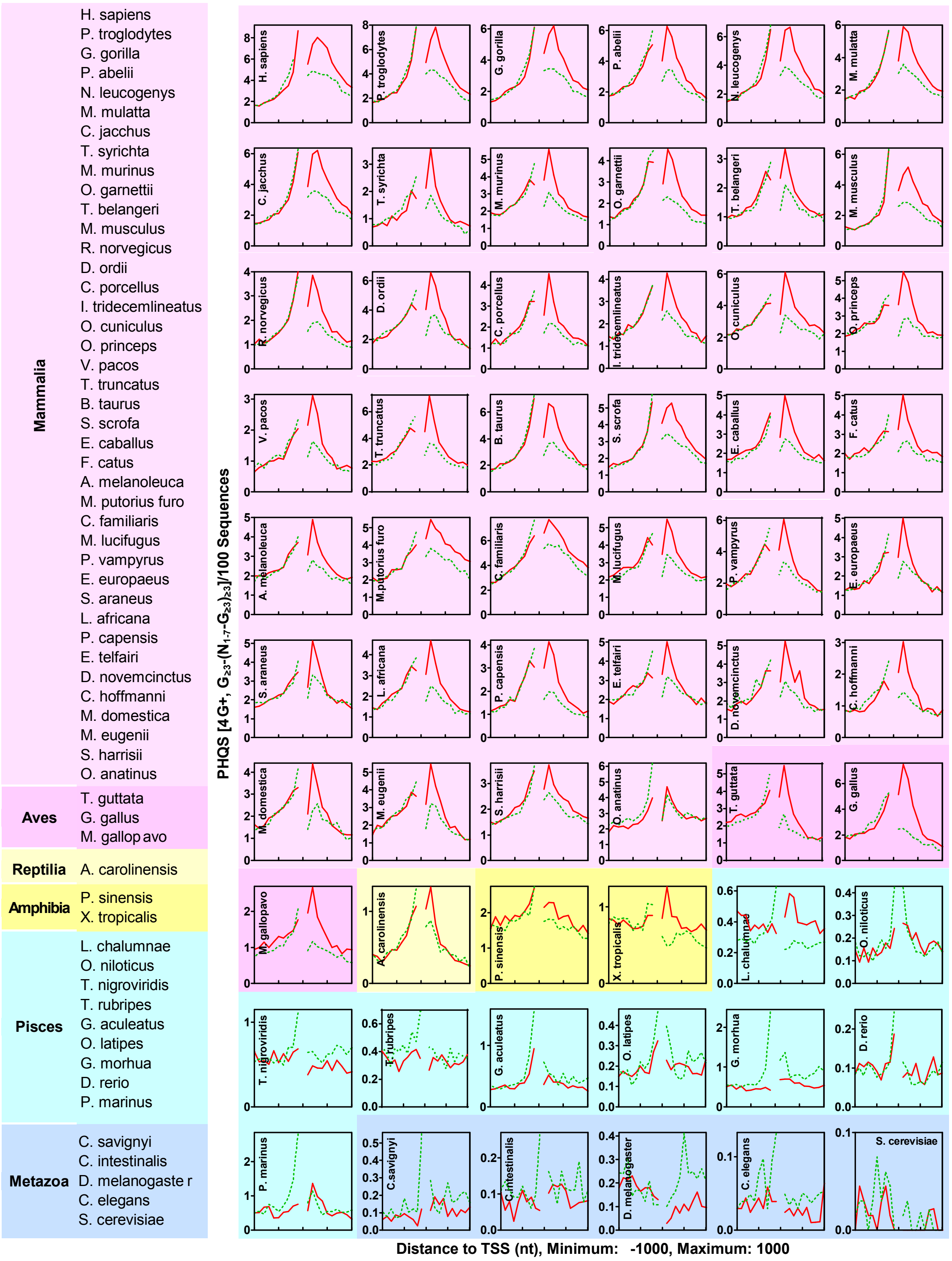

Figure S2. Biased selection for PHQS in 60 species in the Ensembl database. Each panel shows the occurrence of PHQS with four or more G-tracts (4G+) in the non-template (red curve) and template (green curve) strands within the ±1 kb region centered at transcription start sites (TSS).
